# AuNP/Magnetic Bead-Enhanced Electrochemical Sensor Toward Dual Saliva Alzheimer’s Biomarkers Detection

**DOI:** 10.3390/s25134088

**Published:** 2025-06-30

**Authors:** Pengcheng Zhao, Jieyu Wang, Hongju Mao, Lin Zhou, Zhenhua Wu, Yunxing Lu, Teng Sun, Jianan Hui, Guowu Ma

**Affiliations:** 1 School of Stomatology, Dalian Medical University, Dalian 116000, China; zhaopc@dmu.edu.cn (P.Z.); wangjy14@dmu.edu.cn (J.W.); 2 State Key Laboratory of Transducer Technology, Shanghai Institute of Microsystem and Information Technology, Chinese Academy of Sciences, Shanghai 200050, China; hjmao@mail.sim.ac.cn (H.M.); zhoulinzlw@mail.sim.ac.cn (L.Z.); wuzhx@mail.sim.ac.cn (Z.W.); 3 Center of Materials Science and Optoelectronics Engineering, University of Chinese Academy of Sciences, Beijing 100049, China; 4 School of Science and Technology, Shanghai Open University, Shanghai 200433, China; luyunxing@sou.edu.cn; 5 Lin Gang Laboratory, Shanghai 201306, China; sunteng370@lglab.ac.cn; 6 Shanghai Frontier Innovation Research Institute, Shanghai, 201108, China

**Keywords:** electrochemical sensor, Alzheimer’s disease, immunomagnetic beads, gold nanoparticles

## Abstract

Alzheimer’s disease (AD) early screening requires non-invasive, high-sensitivity detection of low-abundance biomarkers in complex biofluids like saliva. In this study, we present a miniaturized, silicon-based electrochemical sensor for sequential detection of two AD salivary biomarkers, lactoferrin (Lf) and amyloid β-protein 1-42 (Aβ_1-42_), on a single reusable electrode. The sensor features a three-electrode system fabricated by sputter-coating a quartz substrate with gold (Au) sensing electrodes, which are further modified with gold nanoparticles (AuNPs) to form 3D dendritic structures that enhance surface area and electron transfer. To improve specificity, immunomagnetic beads (MBs) are employed to selectively capture and isolate target biomarkers from saliva samples. These MB–biomarker complexes are introduced into a polydimethylsiloxane chamber aligned with Au sensing electrodes, where a detachable magnet localizes the complexes onto the electrode surface to amplify redox signals. The AuNPs/MBs sensor achieves detection limits of 2 μg/mL for Lf and 0.1 pg/mL for Aβ_1-42_, outperforming commercial ELISA kits (37.5 pg/mL for Aβ_1-42_) and covering physiological salivary concentrations. After the MBs capture the biomarkers, the sensor can output the result within one minute. Cyclic voltammetry (CV) and electrochemical impedance spectroscopy (EIS) measurements confirm enhanced electron transfer kinetics on AuNP-decorated surfaces, while linear correlations (R^2^ > 0.95) validate quantitative accuracy across biomarker ranges. The compact and integrated design eliminates reliance on bulky instrumentation and enables user-friendly operation, establishing a promising platform for portable, cost-effective AD screening and monitoring.

## 1. Introduction

Alzheimer’s disease (AD), a neurodegenerative disorder (NDD), is characterized by progressive cognitive decline. Typical clinical manifestations include deteriorating cognitive function and behavioral impairments in the elderly and pre-elderly. AD poses significant challenges to public health and social care systems. It features an extended preclinical phase, during which asymptomatic biological changes occur [1]. Monitoring these changes may provide critical insights into disease progression, enabling early clinical interventions to mitigate AD-related harm. However, conventional diagnostic methods for AD, such as neuroimaging and cerebrospinal fluid (CSF) biomarker analysis, often lack the capability for pre-symptomatic detection [2]. While techniques like positron emission tomography and magnetic resonance imaging can identify Aβ plaques, tau tangles, and brain atrophy [3,4], these approaches face significant limitations. They are frequently characterized by high invasiveness (e.g., painful and risky lumbar puncture for CSF sampling), substantial cost, limited accessibility, inadequate sensitivity/selectivity, and an inability to reliably detect the disease early [5]. Therefore, they are not appropriate for AD screening in early clinical stages. Thus, developing non-invasive biosensor diagnostic methods with high sensitivity is imperative. Advances in genomics, proteomics, and metabolomics have positioned saliva as a promising biofluid for detecting disease biomarkers [6]. Often referred to as the “mirror of systemic health”, saliva offers not only a non-invasive sampling route but also biological relevance in AD. AD patients frequently exhibit autonomic dysfunction, a key regulator of salivary secretion [7]. Furthermore, specific components from serum and CSF can enter saliva via osmotic gradients or carrier-mediated transport, resulting in characteristic alterations in AD-related biomarker expression [8,9]. These mechanisms underscore saliva’s potential to reflect neurological pathology and monitor AD progression. Notably, recent studies highlight the role of the oral–brain axis in AD pathogenesis. As the oral cavity is one of the most complex microbial ecosystems in the human body, its inflammatory state may release pro-inflammatory cytokines and pathogen-associated molecular patterns, triggering systemic inflammation and neuroinflammation via blood–brain barrier disruption [10]. This oral–systemic link further supports the use of saliva in AD diagnostics.

Saliva-based detection of AD biomarkers holds significant potential for point-of-care testing (POCT) applications in both diagnosis and disease progression monitoring. Well-characterized AD biomarkers, such as amyloid β-protein 1-42 (Aβ_1-42_), have been found at detectable levels in saliva, with concentrations correlating to AD onset and progression [11]. Furthermore, systemic infections have been identified as a risk factor for AD [12], while aging-associated low-grade inflammation and declining protective immune function synergistically exacerbate AD pathogenesis. As the first line of defense in the immune system, saliva contains antimicrobial proteins whose concentrations reflect innate immune integrity and systemic infection risks, positioning these proteins as candidate biomarkers for AD identification and diagnosis. Lactoferrin (Lf), an iron-binding protein with immunomodulatory activity, demonstrates altered expression in early-stage AD [13,14]. Lf levels correlate with cortical Aβ burden, structural integrity, and memory decline [15], making it a compelling salivary AD biomarker. The combination of Aβ_1-42_ (reflecting amyloid accumulation) and Lf (indicating immune dysregulation) provides a multidimensional strategy for early risk prediction, post-onset diagnosis, and longitudinal monitoring of AD progression. This dual-biomarker approach integrates pathological and immunological axes to address the heterogeneity of AD pathology.

Despite its diagnostic potential, saliva-based detection faces technical challenges. POC devices need to be compact while maintaining high sensitivity and selectivity to detect AD-related biomarkers at ultralow concentrations (e.g., Aβ_1-42_ at pg/mL levels). Immunomagnetic beads (MBs), functionalized with target-specific antibodies (e.g., anti-Aβ_1-42_ or anti-Lf), have proven effective in isolating biomarkers from complex biofluids with high selectivity [16]. This approach minimizes matrix interference and enhances assay specificity and stability, as demonstrated in various biosensor applications [17]. To date, several novel analytical methods have been proposed for the detection of Aβ_1-42_ and Lf, including surface plasmon resonance spectrometry [18], surface-enhanced Raman spectroscopy [19,20], mass spectrometry [21], chemiluminescence [22], imaging-based sensing [23] and electrochemical sensing [24,25,26,27]. Nevertheless, these techniques possess inherent limitations; for instance, equipment dependency and cost-effectiveness constraints may impede their adaptation to point-of-care testing (POCT). Among these methods, electrochemical methods exhibit notable advantages including rapid response, high sensitivity, ease of miniaturization, low cost, and substantial clinical potential [24,25,26,27,28,29]. These characteristics position the technique as a promising alternative for current AD diagnostics [30]. While such sensors have been applied to serum-based AD biomarkers [31] and salivary diagnostics for other diseases [32], their adaptation to AD salivary biomarkers remains limited. Also, the combination of saliva’s intricate composition and low biomarker abundance necessitates improved sensitivity and robustness.

To address these challenges, we developed a miniaturized, high-sensitivity electrochemical sensor that combines MBs-based isolation of AD-related biomarkers with gold nanoparticle (AuNP)-modified electrodes for highly sensitive signal readout. AuNP-modified electrodes showed 3D dendric nanostructures that enhance conductivity and facilitate electron transfer at the sensing surface. Following sample loading into a polydimethylsiloxane (PDMS) chamber, the MB–biomarker immunocomplexes are localized onto the electrode surface using an external magnet, thereby amplifying the detection signal. This system achieves rapid dual-biomarker detection (Aβ_1-42_ and Lf) within 1 min of introducing the MBs, and its compact design is suitable for POC applications, offering significant improvements in diagnostic accessibility, speed, and cost-effectiveness.

## 2. Materials and Methods

### 2.1. Materials

MBs and MB coupling kit were purchased from Suzhou Nanomicro Technology Co., Ltd. (Suzhou, China). Lf and Aβ_1-42_ capture antibodies, biotinylation kit, Lf and Aβ_1-42_ detection antibodies, rabbit anti-goat IgG H&L (Alexa Fluor^®^ 488), goat anti-rabbit IgG H&L (Alexa Fluor^®^ 488), goat anti-rabbit IgG H&L (HRP), and Aβ_1-42_ ELISA kit were purchased from Abcam (Cambridge, UK). Phosphate-buffered saline (PBS) and Tween-20 were purchased from Sangon Biotech Co., Ltd. (Shanghai, China). Bovine serum albumin (BSA) and horseradish peroxidase (HRP) were purchased from Sigma-Aldrich (St. Louis, MO, USA). Adhesion promoter was purchased from Kayaku (Westborough, MA, USA). Photoresist and Developer were purchased from Merck (Darmstadt, Germany). Acetone, FeCl_3_, KCl/HCl, H_2_SO_4_, HAuCl_4_, H_2_O_2,_ and [Fe(CN)_6_]^4−/3−^ were purchased from Shanghai Lingfeng Chemical Reagent Co., Ltd. (Shanghai, China). Polydimethylsiloxane (PDMS) was purchased from Dow Corning (Midland, MI, USA). 3,3′,5,5′-Tetramethylbenzidine (Ultra TMB) and 1,4-Dihydroxybenzene (HQ) were purchased from Shanghai Titan Scientific Co., Ltd. (Shanghai, China).

### 2.2. Immunomagnetic Beads Modification and Assay

To align with the physiological concentrations of biomarkers in saliva, MBs require tailored specific surface areas for optimal detection capabilities. Accordingly, 3 μm beads were employed to target the lower physiological range of Aβ_1-42_ (2–7 pg/mL) [33,34], leveraging their high surface-area-to-volume ratio for increased antibody immobilization and enhanced detection sensitivity. Conversely, 10 μm beads were implemented for Lf detection to accommodate its higher concentration range (5–10 μg/mL) [14]. The moderated surface area of larger beads prevents signal saturation while maintaining assay robustness. To reduce cross-reactivity, the MBs were modified with specific capture antibodies for their respective biomarkers. For streptavidin-modified MBs, capture antibodies were first biotinylated using a commercial biotinylation kit. Subsequently, 20 μL of streptavidin MB suspension (20 mg/mL) was resuspended in 200 μL of 0.05% PBS-T (phosphate-buffered saline with 0.05% Tween-20) and washed twice via magnetic separation (10 s each). After supernatant removal, 100 μL of biotinylated antibody solution was added to the MBs, followed by rotational incubation at room temperature for 1 h. The MBs were then washed twice with 0.1% PBS-T and collected using a magnetic rack. To block nonspecific binding sites, 200 μL of 5% bovine serum albumin (BSA) solution was added (the optimal blocking agent for the MBs was determined in advance through experiments; results shown in Figure S1), and the mixture was incubated under rotation for 30 min at room temperature. Finally, the MBs were washed three times with PBS-T and resuspended in 200 μL of 0.05% PBS-T containing 1% BSA and 0.05% Tween-20, then stored at 4 °C until use. Carboxyl-functionalized MBs were conjugated with antibodies using the manufacturer’s recommended coupling kit, followed by blocking with 5% BSA to minimize nonspecific interactions.

To validate the successful functionalization of capture antibodies on the MBs, an antibody conjugation efficiency assay was designed. Specifically, 5 μL of each type of functionalized MBs (targeting Aβ_1-42_ and Lf, respectively) was separated using a magnetic rack, and the supernatant was removed. Next, 5 μL of fluorophore-conjugated secondary antibodies (diluted 5-fold), species-specific to the primary capture antibodies, was added. The mixtures were incubated under rotation in light-protected conditions at room temperature for 30 min. After incubation, the supernatant was discarded, and the MBs were washed three times with 0.1% PBS-T. The washed MBs were resuspended in 50 μL of 0.05% PBS-T. For fluorescence intensity quantification, 5 μL of the MBs suspension was pipetted onto a glass slide, covered with a coverslip, and imaged using an inverted fluorescence microscope. Excitation at 488 nm was applied, and both bright-field and fluorescence images were captured under a 40× objective. The fluorescence exposure time was set to 3 s. Mean fluorescence intensity of the MBs was analyzed using ImageJ software (version 1.53a).

### 2.3. Fabrication of Quartz Substrate-Based Three-Electrodes System

This study employed optimized magnetron sputtering and lithography protocols to fabricate high-precision chromium/gold (Cr/Au, 30/50 nm) electrode arrays on 4-inch quartz substrates. Prior to deposition, organic contaminants were removed from the quartz surface via oxygen plasma ashing (5 min), generating hydroxyl (-OH) groups and nanoscale roughness to enhance photoresist adhesion. The lift-off process commenced with spin-coating an MCC Primer 80/20 adhesion promoter (2000 rpm, 10 s), followed by AZ4620 photoresist application (500 rpm for 10 s, ramped to 2500 rpm for 30 s). Pre-baking (105 °C, 3 min) preceded UV exposure (45 s, SUSS MA6 UV lithography system). Development in AZ400K:H_2_O (1:3 *v*/*v*) for 120 s yielded patterned photoresist. A 30/50 nm Cr/Au bilayer was deposited via magnetron sputtering, with excess metal and photoresist removed by acetone immersion (60 s), leaving precisely patterned Cr/Au electrodes. For the silver (Ag) layer, an identical lift-off process was applied, with 180 nm Ag deposited via electron beam evaporation. Post-deposition, the Ag electrode was chlorinated by 10 μL of 0.1 M FeCl_3_ (1 min immersion), followed by rinsing with 0.1 M KCl/HCl (2–3 cycles) to form an Ag/AgCl reference electrode. Laser cutting ensured crack-free isolation of individual three-electrode units, preventing electrolyte leakage and maintaining structural integrity. The final electrodes exhibited exceptional surface flatness and pattern fidelity, critical for long-term electrochemical stability.

### 2.4. Fabrication of PDMS Liquid Loading Wells

To facilitate MBs loading, substrate addition, and washing on the electrochemical electrode surface, PDMS loading wells were fabricated. PDMS prepolymer was prepared by mixing the base polymer and curing agent at a 10:1 (*w*/*w*) ratio. The mixture was poured onto a silicon wafer and cured at 120 °C for 20 min. The solidified PDMS layer (~0.5 cm thick) was carefully peeled off and cut into 1 × 1 cm squares. Loading wells (0.8 cm diameter) were created using a punch tool. Prior to bonding, both the PDMS and quartz electrode surfaces were plasma-treated (30 s) to generate -Si-OH groups, enhancing adhesion. The activated surfaces were immediately aligned and pressed together, followed by thermal bonding on a 120 °C hotplate for 15 min to ensure irreversible sealing.

### 2.5. Electrochemical Deposition of AuNPs and Characterization

The PDMS-bonded quartz/gold electrodes underwent electrochemical cleaning and AuNP deposition prior to final testing. For cleaning, 30 μL of 0.1 M H_2_SO_4_ was dispensed onto the electrode surface, followed by 30 cyclic voltammetry (CV) scans (0 to 1.6 V, 100 mV/s) to remove surface oxides and organic contaminants. This process also regenerated electroactive sites via gold oxidation–reduction cycles. Subsequently, AuNPs were electrodeposited on the working electrode via chronoamperometry (CA) (−0.4 V,60 s, 120 s or 180 s) in 0.1 M HAuCl_4_/H_2_SO_4_.

A Dimension Icon (Bruker, MA, USA) atomic force microscope (AFM) was used to analyze the topography of the samples in tapping mode. The images were captured in ambient conditions (T = 24–28 °C) using silicon cantilevers with a nominal tip radius of 10 nm (rtespa-300, Bruker, MA, USA). The scanning rate and the amplitude setpoint were 1 Hz and 240 mV. The reported values for the root-mean-square roughness (σ, the standard deviation of height features) and the surface area ratio (Sdr, the increment of the interfacial surface area relative to the area of the projected flat plane) were calculated from 5 µm × 5 µm AFM images.

Electrochemical impedance spectroscopy (EIS) measurements on AuNP-modified electrodes were carried out using IM6EX system (Zahner, Germany) with the same three-electrode configuration in deionized water containing 5 mmol · L^−1^ [Fe(CN)_6_]^4−/3−^. The EIS measurements were carried out in the range between 2000 Hz and 2 Hz at an open-circuit potential. CV was recorded in the potential range between −0.4 and 0.6 V at the scan rate of 10 mV·s^−1^ in PBS or HQ/H_2_O_2_.

### 2.6. Electrochemical Detection

The operational principle of the electrochemical sensor relies on HRP immobilized via sandwich immunoassay on MBs, which catalyzes substrate redox reactions to induce electron transfer at the electrode interface, generating measurable current signals. To validate this mechanism and assess sequential detection performance, titration experiments were conducted using serially diluted HRP solutions. The bare gold electrode, the AuNP-modified electrode, and the poly(3,4-ethylenedioxythiophene) (PEPOT) modified electrode were tested respectively. After introducing 100 μL of substrate solution into the sensor’s PDMS loading chamber, CA was initiated. Upon current stabilization, 10 μL aliquots of gradient-diluted HRP solutions (80×, 40×, 20×, 10×, 5×) were sequentially introduced, and current signals were recorded for 120 s post-addition.

For Aβ_1-42_ detection, 50 μL anti-Aβ_1-42_ MBs (3 μm, 1 mg/mL) were incubated with 100 μL sample (37 °C, 1 h), washed, then mixed with HRP-detection antibody (4 μg:1 mg beads) under dark rotation (30 min) to form immunocomplexes, finally resuspended in 5 μL PBS. For Lf, 50 μL anti-Lf beads (10 μm, 1 mg/mL) underwent identical sample incubation/washing and received detector antibody (4 μg:1 mg), then 10 μL 100× diluted goat anti-rabbit HRP (RT, dark, 30 min) before final resuspension in 5 μL PBS. The MBs solution (10 mg/mL) was subsequently pipetted into the PDMS well, and a magnet was affixed beneath the working electrode to concentrate MBs. Post-supernatant removal, 100 μL of PBS-based substrate solution with 1 mM HQ and 1 mM H_2_O_2_ was introduced. A customized circuit comprising a differential pulse voltammetry (DPV) module, analog-to-digital converter (ADC, 16-bit resolution), digital-to-analog converter (DAC), and microcontroller unit (MCU) applied a constant potential of −0.1 V (vs. Ag/AgCl). Current signals were recorded at a sensitivity of 1 × 10^−5^ A/V and processed by the MCU for baseline correction and noise filtering. Data were transmitted via USB to a computer for real-time visualization and storage. Optionally, a miniature Bluetooth-enabled electrochemical module (8 × 3 × 1 cm^3^) could be integrated for wireless data streaming to a smartphone application, enabling cloud-based result tracking and remote diagnostics.

## 3. Results

### 3.1. Electrochemical Sensor Design

**Figure 1:**
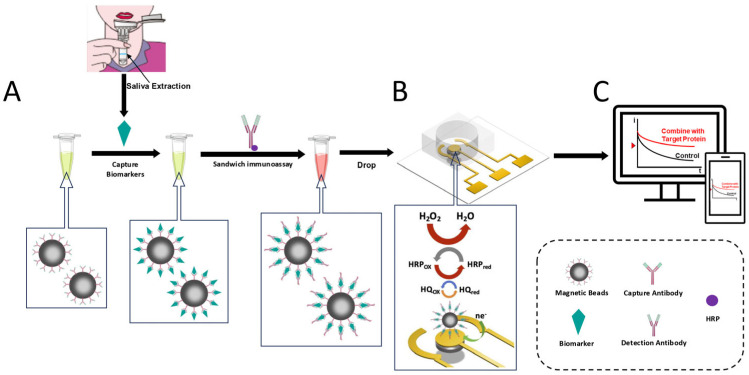
The schematic process using gold nanoparticles and magnetic beads (AuNPs/MBs) enhanced electrochemical sensor for dual Alzheimer’s biomarker detection in saliva. The sensor is mainly composed of a polydimethylsiloxane (PDMS) loading well, a three-electrode structure, a quartz substrate, and a magnet beneath it. (**A**) MBs capture target markers; (**B**) Load MBs into loading wells to generate signals; (**C**) Read the signals.

The schematic of the electrochemical sensor is illustrated in Figure 1. A three-electrode sensor is fabricated on a quartz substrate. Quartz, chosen for its chemical inertness, mechanical robustness, and optical transparency, serves as an ideal platform for miniaturized electrochemical sensors. The substrate is chemically bonded to PDMS loading wells, ensuring leak-free integration.

The MBs modified with corresponding Aβ_1-42_ and Lf capture antibodies are first used to isolate AD-related salivary biomarkers from biofluid. Then, the HRP functionalized detection antibodies are added to generate sandwiched MB–biomarker–HRP complexes. After loading the MBs into the PDMS microwells, the MBs are attracted to the sensor surface by aligned magnets. The HRP-functionalized MBs interact with the substrate within the wells, and redox reactions generate current signals proportional to target concentrations. These signals are transmitted via a plug-in connector to a compact potentiostat for data acquisition and analysis. To optimize sensitivity, AuNPs are deposited on the working electrode via chronopotentiometry [35]. The high surface area and superior conductivity of AuNPs enhance catalytic activity and electron transfer kinetics, significantly improving detection limits. Additionally, a detachable magnet (matching the electrode’s surface area) is integrated beneath the working electrode to concentrate MBs, reducing diffusion distances and amplifying redox-generated signals.

The designed electrochemical sensor employs MBs for biomarker enrichment prior to electrode loading. Compared to studies directly functionalizing electrodes with antibodies [31,36], this approach significantly avoids direct contact between the sample to be tested and the electrode. It not only maintains the cleanliness and stability of the electrode structure but also effectively reduces non-specific adsorption and background noise. Simultaneously, it leverages the high surface-area-to-volume ratio of micron-scale MBs to enhance both sensitivity and specificity. Furthermore, a detachable magnet positioned beneath the quartz substrate—aligned with the working electrode—enables rapid concentration of MBs onto the electrode surface. This localization confines redox reactions directly above the working electrode, stabilizing detection signals and accelerating signal acquisition.

### 3.2. MB Modification Validity Verification

**Figure 2:**
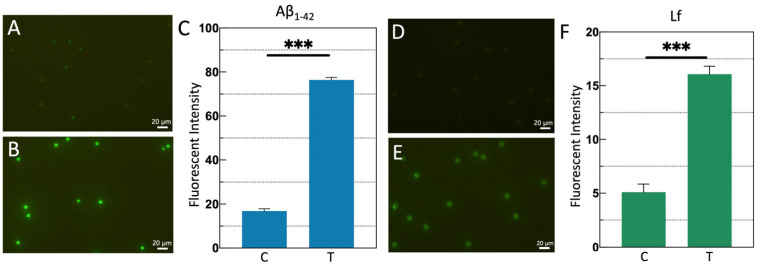
Results of MB functionalization assay. (**A**,**B**) A typical fluorescence field of view of 3 μm MBs before and after modification; (**C**) the normalized fluorescence intensity; (**D**,**E**) a typical fluorescence field of view of 10 μm MBs before and after modification; (**F**) the normalized fluorescence intensity. Both functionalized MB types exhibited significantly higher fluorescence intensities compared to the unmodified control group, with statistically meaningful differences (*p* < 0.001). All assays were performed in triplicate, with data presented as mean ± standard error (SE). Statistical significance was determined using Student’s *t*-test (*** *p* < 0.001).

To confirm successful antibody conjugation, MBs are probed with species-matched fluorescent secondary antibodies specific to the capture antibody host. Figure 2A,B display representative fluorescence fields of view for 3 μm MBs before and after functionalization of Aβ_1-42_ capture antibody, respectively. Figure 2C presents the normalized mean fluorescence intensity of all beads, demonstrating that the functionalized 3 μm MBs exhibit significantly higher fluorescence intensity than the unmodified control (*p* < 0.001). This confirms successful conjugation of capture antibodies onto the 3 μm MB surfaces. Similarly, Figure 2D,E show representative fluorescence fields of view for 10 μm MBs before and after functionalization of the Lf capture antibody, while Figure 2F illustrates the normalized mean fluorescence intensity, validating the effective modification of 10 μm MBs. Additionally, the MBs functionalized for Aβ_1-42_ capture exhibited higher fluorescence intensity compared to those targeting Lf, indicating a greater density of surface-bound capture antibodies on the Aβ_1-42_-specific MBs. This higher antibody loading renders them particularly suitable for detecting ultralow concentrations of Aβ_1-42_, aligning with its physiological range in saliva (2–7 pg/mL).

**Figure 3:**
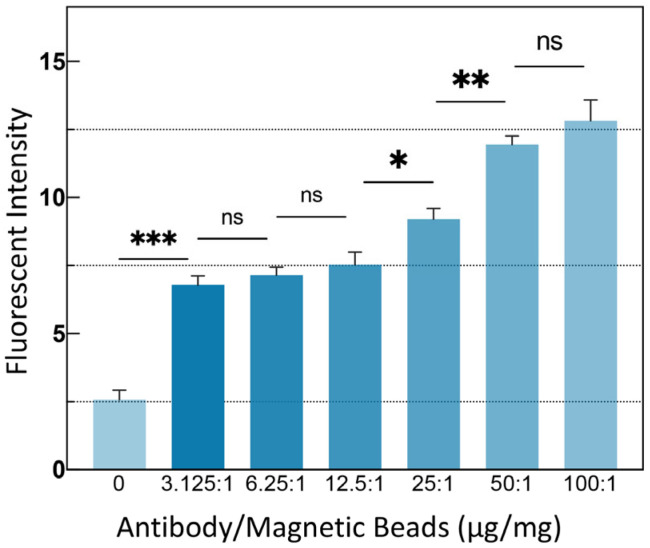
Investigation of the optimal ratio of antibody to MBs for modification. Results show that the optimal ratio is 50 μg antibody to 1 mg MBs. All measurements were performed at least three times, and the data are displayed as mean ± SE. Significance was analyzed using Student’s *t*-test. (*** *p* < 0.001; ** *p* < 0.01; * *p* < 0.05; ns *p* > 0.05).

To achieve optimal detection sensitivity, theoretically, the MBs surface should be conjugated with sufficient antibodies. To determine the ideal coating concentration, 10 μm MBs were coated with gradient-diluted antibodies and characterized using fluorescent secondary antibodies, with the average fluorescence intensity of the MBs representing the amount of successfully coated antibodies. The results, shown in Figure 3, indicate that when the antibody-to-MB ratio was 50 μg: 1 mg, the binding capacity of the MBs reached saturation. This ratio was used for MB coating in subsequent experiments.

### 3.3. Fabrication of the Electrochemical Sensor

**Figure 4:**
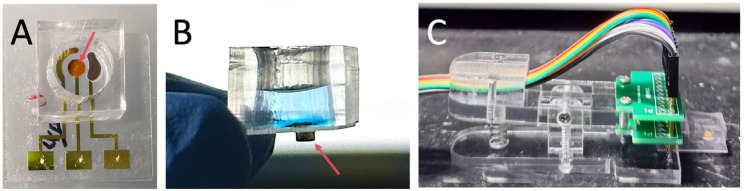
Physical pictures of sensor. (**A**) The arrow indicates the working electrode where AuNPs are deposited. (**B**) Side view of the sensor during detection. The magnet indicated by the arrow can enrich MBs on the surface of the working electrode. (**C**) When in use, the gold fingers on the right side are connected to the electrodes of the sensor, and the back end of the connector is connected to the electrochemical workstation.

The fabricated electrochemical sensor and its associated components are shown in Figure 4. Figure 4A (top view) illustrates the sensor’s three-electrode system encapsulated within a PDMS loading well. The well’s optimized volume ensures complete coverage of the electrode’s active area while minimizing reagent waste. The electrodes extend beyond the PDMS well for seamless connection to the potentiostat via a plug interface. Figure 4B (side view) highlights a detachable neodymium iron boron (NdFeB) magnet positioned beneath the working electrode to concentrate MBs, amplifying electrochemical signals. Post-measurement, the magnet is removed, and the electrode is regenerated through rinsing and drying, enabling repeated use for subsequent samples

Due to the use of MBs for pre-capturing biomarkers, compared with sensors using electrode modification methods [37,38], it avoids complex steps such as electrode immune modification and blocking, making the sensor easier to fabricate, store, and reuse. After completing the test of one sample, the magnet on the back can be removed, and the sensor can be simply rinsed and dried to continue testing the next sample. Subsequent biomarker measurements were performed sequentially on the same sensor. Due to the contamination-free electrode surface, the sensor maintained excellent reproducibility across multiple detections. This significantly increases the cost-effectiveness of the sensor.

### 3.4. Modified Electrode Surface Morphology Characterization

**Figure 5:**
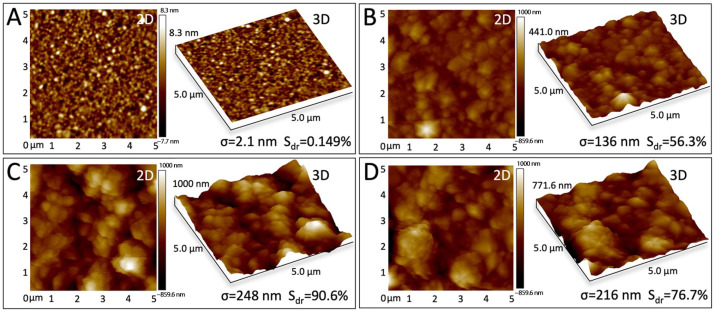

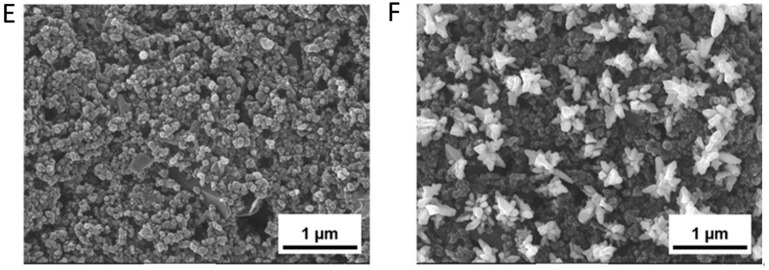
Characterization results of electrode modification under different conditions. Atomic force microscopy (AFM) topographic images of (**A**) bare electrode and electrodes after AuNPs deposition for (**B**) 60 s, (**C**) 120 s, (**D**) 180 s. Scanning electron microscopic (SEM) images of gold sensing surface (**E**) before and (**F**) after 120 s potentiostatic deposition of AuNPs.

AuNPs functionalization enhances sensor sensitivity by increasing the working electrode’s specific surface area and reducing electrical resistance, thereby amplifying current response. To optimize this modification, we compared three deposition durations (60 s, 120 s, 180 s) for their morphological impacts. Figure 5A–D presents typical AFM topographical images of the electrode surfaces after AuNPs deposition for different durations. The corresponding roughness parameters σ (representing Rq, the root means square roughness) and Sdr (the developed interfacial area ratio) obtained from the AFM images are listed below the corresponding pictures. The results show that compared to the bare gold electrode (Figure 5A), the AuNP-modified electrodes (Figure 5B–D) develop numerous larger-diameter protrusions, with both σ and Sdr increasing significantly. This indicates an enlarged contact area between the electrode and the electrochemical substrate, which can enhance sensor sensitivity. The AuNPs deposition time significantly influences the surface morphology. As the deposition time increases from 60 s (Figure 5B) to 120 s (Figure 5C), the protrusions begin to aggregate into clusters, and the values of both σ and Sdr increase. However, when the time is further extended to 180 s (Figure 5D), excessive aggregation occurs, leading to a decrease in both σ and Sdr values. Consequently, the AFM results demonstrate that the AuNPs deposited for 120 s exhibit optimal roughness (σ) and Sdr values. Figure 5E,F present scanning electron microscopy (SEM) images of bare gold electrodes versus AuNP-modified electrodes after 120 s deposition, respectively. The functionalized surface exhibits optimally dense 3D dendritic nanostructures compared to the unmodified electrode.

### 3.5. Modified Electrode Electrochemical Characterization

**Figure 6:**
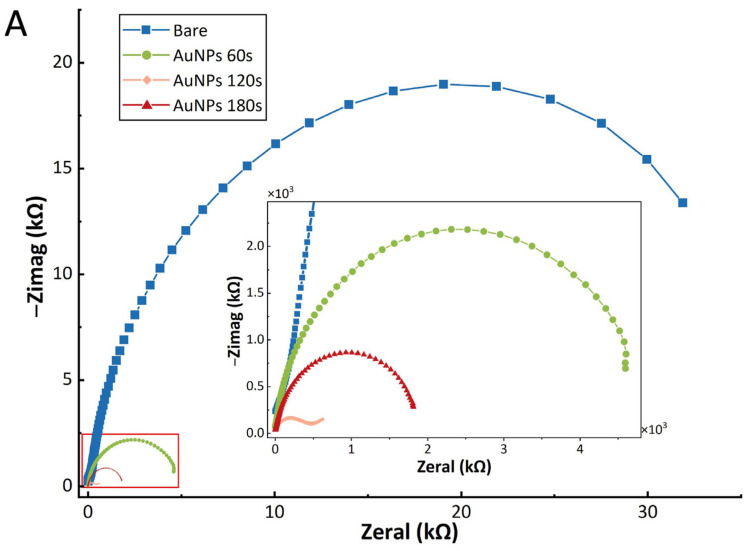

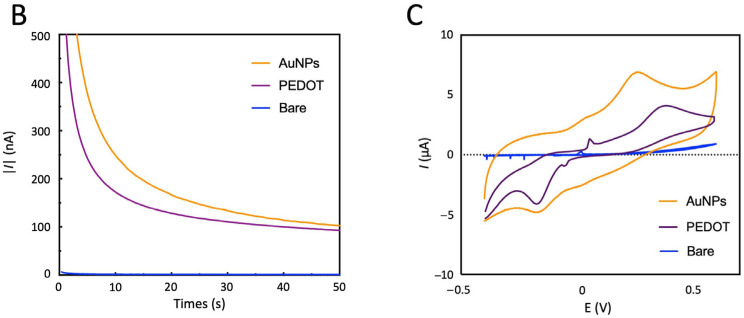
Electrochemical characterization results of the modified electrode. (**A**) Electrochemical impedance spectroscopy (EIS) of different electrodes recorded in deionized water containing 5 mmol·L^−1^ [Fe(CN)_6_]^4−/3−^. (**B**) Chronoamperometry (CA) results comparing the AuNP-modified electrode (orange line), poly(3,4-ethylenedioxythiophene) (PEDOT)-modified electrode (purple line), and bare electrode (blue line). The AuNP-modified electrode exhibited a fivefold increase in current response over the recording period. (**C**) Cyclic voltammetry (CV) profiles of the AuNP-modified (orange), PEDOT-modified electrode (purple line) and bare (blue) electrodes. The AuNP-functionalized surface shows the strongest peak currents and the largest peak areas, reflecting significantly increased electroactive site density.

In the Nyquist plot of EIS, the high-frequency region typically exhibits a semicircular feature, corresponding to a kinetically controlled process at the electrode interface, while the linear response in the low-frequency region usually indicates a mass-transfer controlled process [39]. Figure 6A presents the Nyquist plots of EIS for three electrodes with different deposition times of AuNPs, measured using a ferricyanide/ferrocyanide ([Fe(CN)_6_]^4−/3−^) redox probe in deionized water. Compared to the bare gold electrode, the AuNP-modified electrodes show smaller semicircle diameters in the high-frequency region, signifying reduced electron transfer resistance (Ret). Notably, the electrode modified with AuNPs deposited for 120 s exhibits the smallest Ret. This suggests that the electrode prepared under this deposition condition possesses significantly enhanced electrical conductivity. However, when the deposition time is extended to 180 s, the Ret conversely increases. This increase is likely attributable to the aggregation of AuNPs, which reduces the specific surface area.

The assess the enhanced conductivity of the AuNPs-modified electrodes, CA and CV are conducted on the sensor before and after AuNPs deposition. Also, the comparative testing is conducted using PEDOT-modified electrodes. After adding the electrochemical substrate to the electrochemical sensor loading well, the results demonstrated that the AuNP-modified electrodes exhibited superior CA (Figure 6B) and CV (Figure 6C) responses compared to both bare electrodes and PEDOT-modified counterparts. This indicates that the surface functionalization using AuNPs significantly enhances the electron transfer efficiency of the electrode. Similar to the typical CV curves in other studies that also use AuNP-modified electrodes [40], the superior electrochemical performance may originate from dendritic AuNPs’ dual advantages of expanded surface area and optimized electron transfer kinetics [41]. At the same time, the open, branch-like pores let H_2_O_2_ and reaction products diffuse rapidly, preventing local depletion and enabling larger, steadier catalytic currents [42]. Together, these effects raise signal-to-noise, cut the charge-transfer resistance, and routinely push detection limits of HRP-based amperometric assays down to the ng/mL or even pg/mL range [43].

### 3.6. Electrochemical Sensor Test Results

**Figure 7:**
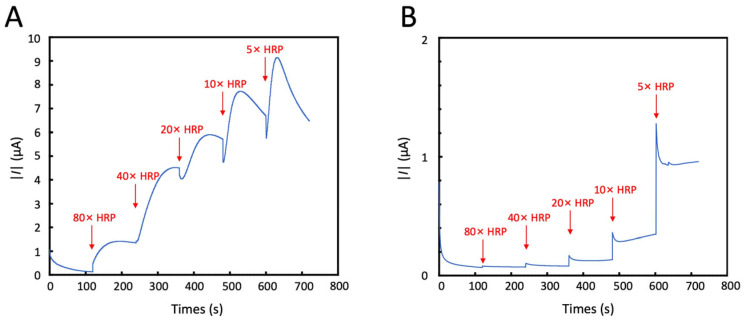
Titration test of the electrochemical sensor. (**A**) AuNPs-modified electrode. (**B**) PEDOT-modified electrode. After adding the substrate to the loading well, the CA test is conducted. The horseradish peroxidase (HRP) solution is gradually diluted and dropped into the loading well at the time indicated by the arrow, with an applied potential E = −0.1 V.

The comparisons of CA responses between the AuNP-modified and the PEDOT-modified electrodes are shown in Figure 7A and 7B, respectively. Experimental results demonstrated immediate current signal changes following each HRP addition, with signal intensity inversely proportional to the dilution factor (i.e., lower dilution ratios yielded stronger currents), collectively exhibiting a stepwise increase. Therefore, when MBs with more biomarkers bound to their surface (which also means more HRP bound) are loaded onto the electrode, the HRP on the surface can catalyze the substrate reaction, generating a change in the current signal that is directly proportional to the number of biomarkers. Meanwhile, the results also indicate that the AuNP-modified electrode display more pronounced current variations than PEDOT-modified electrode, indicating higher detection sensitivity. This enhanced performance renders the AuNP-modified system particularly suitable for detecting low-concentration AD biomarkers in saliva, where target analytes exist at trace levels.

### 3.7. Detect Lf Using Electrochemical Sensors

**Figure 8:**
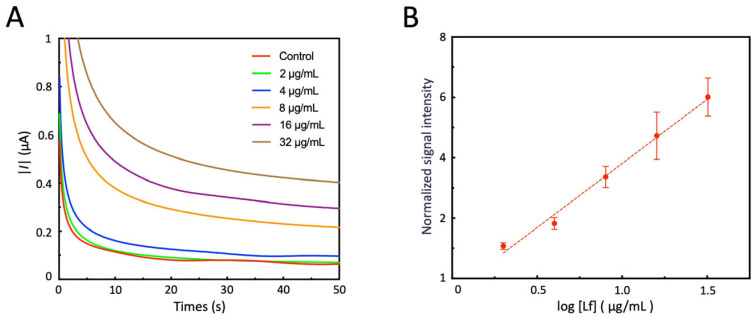
Analysis of lactoferrin (Lf) standard samples using electrochemical sensors. (**A**) A typical result of real-time electrochemical chronocoulometric measurement by the sensor; (**B**) normalized results of repeated measurements and linear fitting. The regression equation of the straight line is y = 4.2457x − 0.4338, with a coefficient of determination of R^2^ = 0.991. Each measurement result is standardized by dividing the chronocoulometric response of the control group. All measurements were conducted at least three times, and the data are presented as the mean ± SE.

The electrochemical sensor is used to detect the gradient diluted Lf standard samples. The typical detection results are shown in Figure 8A. To evaluate the response characteristics of the sensor, the logarithm of the marker concentration (log10 [Lf]) is taken as the independent variable (X), and the normalized sensor signal obtained by dividing the measurement results by the chronoamperometric response of the control group is taken as the dependent variable (Y). A scatter plot is drawn, and a linear regression analysis is performed (Figure 8B). All three detections are performed consecutively. After completing the measurement of one sample, the electrode surface is rinsed and thoroughly dried before proceeding to the next sample. The results show that within the range of 2 μg/mL to 32 μg/mL, the signal has a significant linear relationship with log10 [Lf]. The limit of detection (LOD) of the sensor is 2 μg/mL, which meets the detection requirements for the physiological concentration of the target marker. This logarithmic–linear response characteristic is consistent with the typical behavior of electrochemical immunosensors [38], providing a reliable calibration method for subsequent sample detection. The concentration of Lf in the saliva of normal people is about 10 μg/mL, but in AD patients it is about 5 μg/mL [14]. Compared with other electrochemical sensors for detecting Lf [44,45], our sensor has a detection range that is more in line with the physiological concentration of Lf in saliva and is suitable for the detection of saliva from AD patients at their original concentration.

### 3.8. Detection of Aβ_1-42_ by Electrochemical Sensor

**Figure 9:**
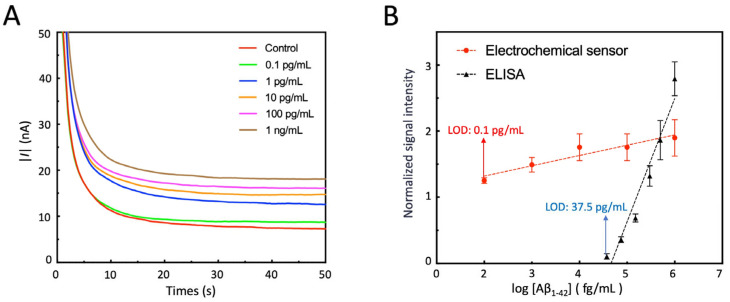
Analysis of Aβ_1-42_ standard samples using electrochemical sensors. (**A**) A typical result of real-time electrochemical chronocoulometric measurement by the sensor; (**B**) normalized results of repeated measurements using electrochemical sensor or commercial ELISA kits and linear fitting. The regression equation of the fitted line for sensor measurement results is y = 0.1556x + 0.995, R^2^ = 0.9618; the regression equation of the fitted line for ELISA kit measurement results is y = 1.8644x − 8.6933, R^2^ = 0.9454. Each measurement result of sensor is standardized by dividing the chronocoulometric response of the control group. All measurements were conducted at least three times, and the data are presented as mean ± SE.

The developed sensor is used to detect the gradient diluted Aβ_1-42_ standard samples. The typical detection results are shown in Figure 9A. To evaluate the response characteristics of the sensor and compare it with the commercially available Aβ_1-42_ ELISA kit, the logarithm of the marker concentration (log10 [Aβ_1-42_]) is taken as the independent variable (X), and the normalized measurement results are taken as the dependent variable (Y). A scatter plot is drawn, and a linear regression analysis is performed (Figure 9B). All three detections are performed consecutively. After completing the measurement of one sample, the electrode surface is rinsed and thoroughly dried before proceeding to the next sample. The results showed that the electrochemical sensor had a linear relationship between the signal and log10 [Aβ_1-42_] within the range of 0.1 pg/mL to 1 ng/mL, with a LOD of 0.1 pg/mL, covering the physiological concentration range of the target marker and meeting the detection requirements for saliva. The ELISA kit had a linear relationship between the signal and log10 [Aβ_1-42_] within the range of 37.5 pg/mL to 1 ng/mL, with a LOD of 37.5 pg/mL, which is higher than that of the electrochemical sensor and did not fully cover the physiological concentration range of the target marker. The concentration of Aβ_1-42_ in the saliva of normal people is around 2 pg/mL, while in AD patients it is about 7 pg/mL [33,34]. Compared to electrochemical sensors employing gold electrodes reported in prior studies [46,47], our approach achieves a significantly lower LOD, rendering it uniquely suited for quantifying trace levels of Aβ_1-42_ in saliva—a biofluid where this biomarker exists at ultralow physiological concentrations.

### 3.9. Test of the Stability and Reproducibility of the Sensor

**Figure 10:**
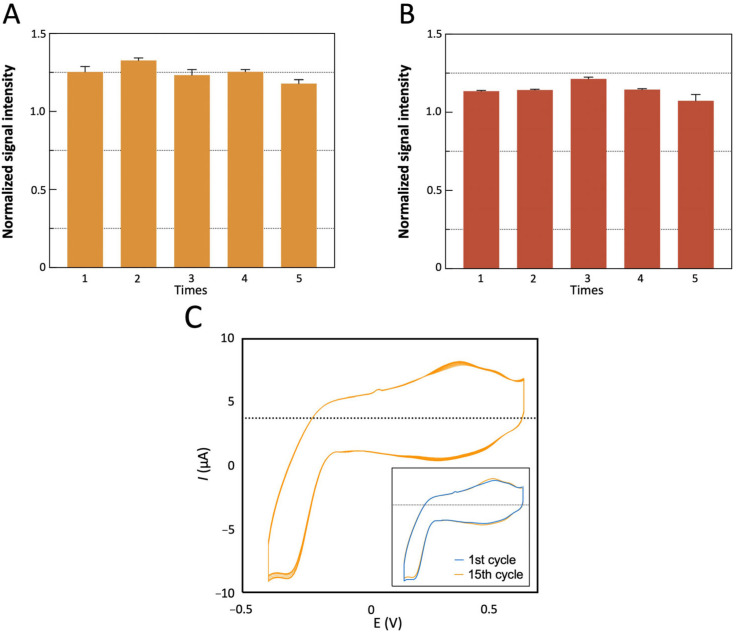
(**A**) Five consecutive detection cycles of 0.1 pg/mL Aβ_1-42_. (**B**) Five consecutive detection cycles of 2 μg/mL Lf. (**C**) Fifteen successive CV cycles in PBS; inset: superimposed initial (cycle 1, blue) and final (cycle 15, yellow).

To enable repeated sample testing, the electrode material must exhibit robust reproducibility and stability [48]. As shown in Figure 10A, the sensor demonstrated excellent detection consistency with 3.29% RSD across five measurements of 0.1 pg/mL Aβ_1-42_. Similarly, as shown in Figure 10B, the sensor demonstrated excellent detection consistency with 3.85% RSD across five measurements of 2 μg/mL Lf. Following multiple usage cycles, 15-cycle CV testing in PBS (Figure 10C) revealed enhanced electrochemical activity, including (i) A 7.49% increase in anodic peak current after 15 cycles (Table 1), attributed to the removal of surface passivation layers during potential scanning; (ii) >95% curve overlap between the initial and final cycles, confirming structural integrity. These results collectively validate the sensor’s operational stability and reusability for repeated biomarker quantification.

## 4. Discussion

While current biosensor research for early AD diagnosis predominantly focuses on CSF and blood, some studies propose POCT-compatible sensor designs. For example, de Oliveira et al. developed a disposable microfluidic platform based on an electrochemical immunosensors [49], demonstrating good concordance with ELISA. In a subsequent study by the same team, they proposed a magnetic immunoassay based on low-cost, screen-printed electrodes [50], making the detection method more aligned with the POCT concept. However, the above-mentioned methods still face challenges regarding invasive sample collection and processing complexity. To address the need for invasive samples, salivary diagnostics for AD patients has recently emerged [51,52]. This approach has garnered significant attention due to the non-invasiveness of saliva collection—which aligns well with the POCT concept—and the high relevance of salivary components to the “oral–brain axis” hypothesis [10]. Therefore, in this study, we developed an electrochemical sensor utilizing AuNPs modification and MBs pre-capture, aiming to achieve highly sensitive and rapid detection of two salivary AD biomarkers, Lf and Aβ_1-42_.

Although saliva possesses significant potential as a POCT sample matrix, its high complexity presents challenges. Key macromolecular components include high-molecular-weight glycoproteins, primarily mucins [53], and other proteins such as secretory immunoglobulin A (sIgA), amylase, and lysozyme. To overcome salivary matrix complexity, researchers have investigated protocols for isolating non-target proteins and carbohydrates from raw saliva to obtain pre-processed samples more amenable to analysis [54,55,56]. Furthermore, salivary composition can be influenced by physiological factors, stress, and circadian rhythms [8,9], necessitating the development of corresponding matrix effect mitigation strategies and standardized sample collection protocols.

In plasma sample detection, sample dilution can be used to mitigate potential interferences and reduce non-specific binding signals; for instance, de Oliveira et al. diluted plasma samples 5.5-fold [50]. However, for saliva, biomarker concentrations are typically lower than those in blood [57,58]. Excessive dilution risks further reducing biomarker levels below the detection limit, increasing detection difficulty. Karaboğa et al. developed a single-use electrochemical sensor capable of detecting the Parkinson’s disease biomarker DJ-1 at femtogram levels in standards and demonstrated its capability using spiked saliva samples [59], albeit limited to single use. Ondrej Zitka et al. utilized microfluidics combined with electrochemistry to detect Lf isolated using MBs [27], achieving a LOD of 0.1 μg/mL for Lf in isolated samples from biological fluids, including saliva, and for standards. Altuntas and Buyukserin reported a biosensor for detecting Aβ_1-42_ with an LOD of 0.5 pg/mL and a linear dynamic range of 0.5 pg/mL to 100 ng/mL [60], testing performance using peptide solutions prepared in artificial saliva. Compared with the previous studies, our approach utilizes a strategy combining MB pre-separation with detection via AuNP-modified electrodes. This methodology avoids contamination and non-specific adsorption risks associated with direct sample loading onto electrodes [61], thus significantly enhancing electrode stability and long-term reusability. While achieving comparable detection limits to other studies (2 μg/mL for Lf and 0.1 pg/mL for Aβ_1-42_), our sensor demonstrates distinct advantages in simplified fabrication, miniaturization potential, and dual-biomarker detection capability. Nevertheless, several issues require resolution before extending our method to real-sample detection.

First, standardized saliva collection protocols must be established. Collection conditions significantly impact biomarker levels. Cui et al. used six methods to collect saliva from AD patients [62], finding that different methods greatly affected the concentration and correlation of Aβ_1-40_, Aβ_1-42_, t-tau, and p-tau. Standardization of saliva collection is therefore crucial to minimize intra- and inter-individual variability. Beyond whole saliva, studying secretions from individual glands is important, as whole saliva contains non-salivary components like exfoliated epithelial cells, bacteria, and gingival crevicular fluid (GCF), which may interfere with detection.

Second, saliva’s high viscosity poses challenges. It limits the use of microfluidic arrays with unprocessed raw saliva and increases the risk of bead aggregation during incubation and washing steps [63]. As described in the methods, MBs require incubation with the sample. During this, saliva’s viscosity heightens the risk of bead aggregation. Since the bead’s target capture efficiency depends on its available surface area and freedom of movement within the sample, aggregation detrimentally affects biomarker capture. Although we employed rotational incubation and could add agitation steps to reduce aggregation, excessive mixing steps were ultimately omitted for simplicity. Mucin MUC5B is the primary contributor to saliva’s high viscosity [64]. To counteract this, future work could explore saliva filtration for pre-processing. Several studies have utilized a commercial saliva collection system [65,66,67]; compared to passive drooling into a tube, this system offers simpler, more hygienic collection and, crucially, yields lower-viscosity saliva while facilitating the removal of dead cells, glycoproteins, and other subcellular material. Farsaeivahid et al. also employed filtration for saliva pre-processing [68], finding that a 20 mg nylon wool (NW) filter reduced viscosity by 80% while only reducing protein content by 6%, making it suitable for a salivary spike (S) protein diagnostic system. NW pre-filtration significantly improved the LOD for salivary S protein detection by fivefold and reduced the relative standard deviation (RSD) twofold compared to unfiltered saliva.

Finally, multiplexed detection of combined biomarkers is crucial for enhancing diagnostic accuracy in early AD detection [69]. AD is multifactorial and polygenic; beyond Aβ pathology and tau-related pathophysiology, numerous other molecular processes and homeostatic networks are impaired [70]. According to the NIA-AA research framework, validated biomarkers reflecting other key AD pathophysiological processes can enrich and expand the AT(N) system into an ATX(N) system [71]. For example, vascular, inflammatory, or synaptic biomarkers could be integrated to create ATV(N), ATI(N), or ATS(N) systems. Given the spatiotemporal interconnections between AD pathophysiological processes (e.g., Aβ and tau synergy), these systems could provide a more comprehensive AD phenotype description. Our study included Aβ_1-42_ and Lf biomarkers, targeting central nervous system amyloid deposition and oral immune microenvironment changes, respectively, reflecting AD pathology from different angles. Future work should incorporate additional biomarkers like miRNAs or metabolites, referencing the “ATX(N)” concept to enhance the biological richness of salivary biomarker panels and improve disease prediction accuracy. Simultaneous detection of multiple biomarkers necessitates sensors with multiple detection units for parallel signal acquisition.

Therefore, to systematically apply our developed sensor to real saliva detection, our next step involves optimizing the sensor’s circuit design and electrode architecture based on the existing principle to develop a multiplexed electrochemical sensor capable of detecting over three biomarkers simultaneously. To achieve this, we plan to modify the existing PDMS sample loading well, integrating multiple detection units with a microfluidic system. Microfluidics will be used to compartmentalize different-sized MBs (with different immunological modifications on their surfaces) into distinct detection zones within the sensor, thereby developing a microfluidic–electrochemical sensor with enhanced automation capabilities.

## 5. Conclusions

This paper designs and develops an electrochemical sensor that uses AuNP modification and immunomagnetic bead pre-capture, aiming to achieve the highly sensitive and rapid detection of two AD salivary biomarkers. The sensor employed sputter-coated Cr/Au electrodes on a quartz substrate, bonded to a miniaturized PDMS microfluidic chamber, enabling on-chip reaction and real-time detection. Electrochemical deposition of AuNPs onto the working electrode resulted in a 3D dendric structure, significantly increasing the density of reactive sites and thereby enhancing sensitivity. CV and CA tests demonstrated markedly improved current responses after AuNPs modification. Functionalized MBs were employed to capture biomarkers from samples before loading onto electrodes. This design avoids unstable electrode modifications, difficulties in sensor reuse, and potential contamination from direct sample contact. A detachable magnet integrated at the working electrode effectively concentrated MBs-biomarker complexes, amplifying redox signals. This achieved a LOD for Aβ_1-42_ superior to commercial ELISA kits within physiological concentration ranges. While current validation used standard solutions, future clinical studies with saliva samples are essential prior to deployment.

In summary, the developed electrochemical sensor, designed for AD saliva detection, incorporates innovative features like MB pre-capture, magnetic enrichment, and a PDMS loading well, conceptually aligning with the requirements for salivary diagnostics. Future developments will build upon the current detection principle to comprehensively enhance the clinical translation potential of the sensing system. This will be achieved by addressing saliva sample collection, pre-processing, and detection system design to mitigate matrix effects, improve multiplexed biomarker detection capability, and automate the assay workflow via microfluidic integration. The ultimate goal is to realize a non-invasive POCT sensor for the early diagnosis of AD.

## Figures and Tables

**Table 1:** Stability parameters of AuNP-modified electrodes.

Number of Cycles	ΔIpa (%)	ΔEpa (mV)	ΔIpc (%)	ΔEpc (mV)	Overlap
1 (ref)	-	-	-	-	-
15	7.49%	−21	2.87%	0	95.96%

## Data Availability

Data are contained within the article and Supplementary Materials.

## Supplementary Materials

The following supporting information can be downloaded at https://www.mdpi.com/article/10.3390/s25134088/s1: Figure S1: Comparison of MB blocking methods.

## Abbreviations

The following abbreviations are used in this manuscript:

AD	Alzheimer’s disease
NDD	Neurodegenerative disorder
CSF	Cerebrospinal fluid
PAMPs	Pathogen-associated molecular patterns
POCT	Point-of-care testing
Aβ_1-42_	amyloid β-protein 1-42
Lf	Lactoferrin
MBs	Immunomagnetic beads
AuNPs	Gold nanoparticles
PDMS	Polydimethylsiloxane
HRP	Horseradish peroxidase
PBS	Phosphate-buffered saline
BSA	Bovine serum albumin
CV	Cyclic voltammetry
DPV	Differential pulse voltammetry
ADC	Analog-to-digital converter
DAC	Digital-to-analog converter
MCU	Microcontroller unit
CA	Chronoamperometry
LOD	Limit of detection
SEM	Scanning electron microscopy
EIS	Electrochemical impedance spectroscopy
Sdr	Surface area ratio
AFM	Atomic force microscope
TMB	3,3′,5,5′-Tetramethylbenzidine
HQ	1,4-Dihydroxybenzene
EIS	Electrochemical impedance spectroscopy
