# Supplementary material for: AuNP/Magnetic Bead-Enhanced Electrochemical Sensor Toward Dual Saliva Alzheimer’s Biomarkers Detection
Source: Sensors (Basel). 2025 Jun 30;25(13):4088. doi: 10.3390/s25134088 (PMC12252058; doi:10.3390/s25134088)
Supplement: Supplementary file 1 [file sensors-25-04088-s001.zip › sensors-3647355-supplementary.pdf]

Comparison of MB Blocking Methods

Initial validation experiments revealed that unmodified control MBs exhibited low fluorescence intensity due to non-specific adsorption (NSA) of fluorescent labels. To mitigate NSA, post-functionalization blocking was performed using three agents: 100 mM ethanolamine, 1% casein, and 5% bovine serum albumin (BSA). These were tested against unblocked functionalized MBs (Unblock) and unmodified MBs (Unmodified), as shown in Figure S1. Functionalized MBs demonstrated significantly higher NSA signals than unmodified controls, confirming NSA predominantly originated from surface-immobilized antibodies. Among blocking agents, 100 mM ethanolamine paradoxically increased NSA (vs. Unmodified), while 1% casein and 5% BSA reduced NSA to levels below those of unmodified MBs. Although no statistically significant difference was observed between casein and BSA ($p>0.05$), 5% BSA was selected for subsequent experiments due to its ease of preparation and storage stability compared to casein.

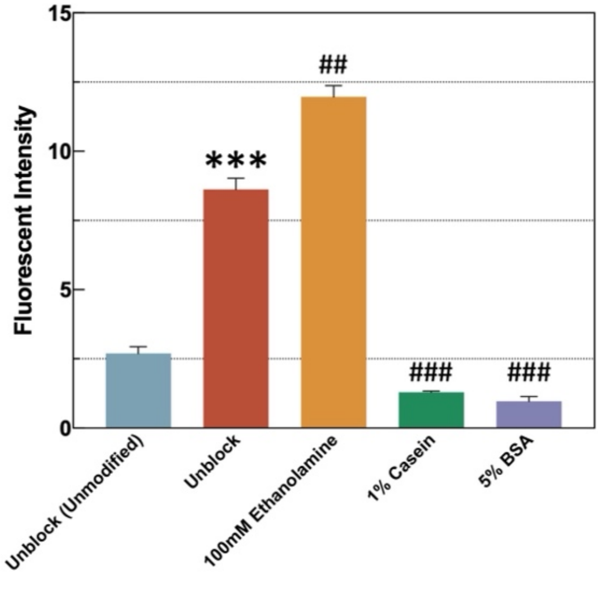

Figure S1. Comparison of MBs blocking methods. Unblocked functionalized MBs (Unblock) exhibited significantly higher non-specific adsorption compared to unmodified MBs (Unmodified). Treatment with 100 mM ethanolamine further increased non-specific signals relative to Unblock MBs. In contrast, 1% casein and 5% BSA significantly reduced non-specific signals below Unmodified MB levels (*p<0.05 vs. Unblock/Unmodified; **p<0.01; **p<0.001; #p<0.05 vs. Unblock; ##p<0.01; ###p<0.001). All experiments were performed in triplicate, with data expressed as mean ± standard error (SE). Statistical significance was analyzed using Student's t-test.
